# Pharmacokinetics of cytisine after single intravenous and oral administration in rabbits

**DOI:** 10.2478/v10102-010-0003-5

**Published:** 2010-03-29

**Authors:** Henri Astroug, Roumiana Simeonova, Lilia V. Kassabova, Nikolay Danchev, Dobrin Svinarov

**Affiliations:** 1 Department of Pharmacology, Pharmacotherapy and Toxicology, Faculty of Pharmacy, Medical University, Sofia, Bulgaria; 2 Central Laboratory of Therapeutic Drug Monitoring and Clinical Pharmacology, Alexander University Hospital, Faculty of Medicine, Medical University, Sofia, Bulgaria

**Keywords:** cytisine, pharmacokinetics, rabbits, HPLC method

## Abstract

The aim of this study is to develop a sensitive HPLC method for the quantitative determination of cytisine in serum and to characterize the pharmacokinetic behaviour of cytisine after oral and intravenous administration in rabbits. The pharmacokinetic behaviour of cytisine is studied in male and female New Zealand rabbits after oral and intravenous administration. Cytisine is administered orally (dose of 5 mg/kg b.w.) under fasting condition (12 hours) and intravenously (dose 1 mg/kg b.w.) in the marginal ear vein. Cytisine serum concentrations are measured using a highly selective and sensitive validated HPLC method with UV detection. Linearity of the method is in the range 12–2 400 µg/L; accuracy and precision are both within ± 10%, and the limit of detection is 4 µg/L. Selectivity and stability are also validated. Basic pharmacokinetic parameters of cytisine after single oral and intravenous administration are calculated using TOPFIT software. Pharmacokinetic analysis suggests a rapid but incomplete absorption of cytisine after oral administration.

## Introduction

Tobacco smoking is the first avoidable cause of deaths and morbidity in developed countries (Mokdad *et al*., 2004). Smoking the leaves of *Cytisus laburnum* L. (golden rain tree) or *Ulex europaeus* is proposed for the treatment of tobacco dependence in 1955 (Lickint, 1956). Tabex^®^ is an original Bulgarian drug of plant origin, intended for treatment of nicotine dependence. The preparation is developed and introduced in 1964 on the basis of the alkaloid cytisine contained in the plant *Laburnum anagyroides* Medic. (*Cytisus laburnum* L.) fam. Fabaceae (Leguminosae) which is distributed in the southern parts of Central Europe and Italy (Dobreva *et al*., 2005). The largest quantity of the alkaloid – up to 3%, is found in the seeds. Cytisine possesses similar mechanism of action to that of nicotine. It excites autonomic nervous ganglia, increases adrenaline release by the adrenal gland, stimulates the locomotor center and elevates arterial blood pressure. Cytisine is readily dissolved in water.

The studies of cytisine pharmacokinetics in laboratory animals are rather old and insufficient. There are reports in literature on defining its pharmacokinetic behaviour after intravenous administration at a dose of 2 mg/kg b.w., and administration as a transdermal therapeutic system to rabbits (Sariev *et al*., 1999). Cytisine concentration in blood is subjected to the laws of an open two-compartmental pharmacokinetic model. Elimination is rapid with a half-life of 0.95 h. The short-term retention of cytisine in the body may be judged also from the mean residence time (MRT – 0.98 h) and total body clearance (Cl_tot_ – 2.4 L/h). Upon determination of kinetic behaviour of cytisine in mice, Klocking *et al*. (Klocking *et al*., 1985) found peak serum concentrations 2 hours after oral administration. The level of absorption is 42%. The highest cytisine concentrations are found in the liver, kidneys and suprarenal glands. Excretion is mainly with urine, where, 24 hours after intravenous and oral administration, 32% and 18% respectively of the dose is recovered. The half-life is 3 hours. Part of the administered cytisine is eliminated with the bile where the concentration of the substance is 200 times higher than blood concentration 3–4 hours after intravenous, and 40 times higher – after oral administration. The concentration of unchanged cytisine in the feces is 3% 6 hours after intravenous and 11% after oral administration (Klocking *et al*., 1985).

The aim of this study is to develop a sensitive HPLC method for the quantitative determination of cytisine in serum and to characterize the pharmacokinetic behaviour of cytisine after oral and intravenous administration in rabbits.

## Materials and methods

Cytisine was kindly supplied by Sopharma Pharmaceuticals, Sofia, Bulgaria (Batch No 20205) (analytical certificate No 51/04.05.2005).

### Quantitative analysis of cytisine in rabbit serum

#### Chemicals and Reagents

All solvents used for the HPLC mobile phase and extraction (water, acetonitrile, methanol, ethyl acetate) are of HPLC grade. All other reagents are obtained from Merck, Darmstadt, Germany, and phosphoric acid (85%) is purchased from Janssen Chimica, Germany. Cytisine is purchased from Sigma, and sulfanilamide obtained from the Bulgarian Drug Agency is used as internal standard. Stock and intermediate standards of cytisine and sulfanilamide are prepared in methanol. Calibration curve (CC) standards and quality controls (QC – low, middle and high) are prepared in advance in serum pools, are aliquoted for daily use, and are kept frozen below −20 °C until analysis. For the preparation of calibration and control samples a blank pool of commercial animal serum based quality control material is used. Calibration and control samples are prepared by mixing of definitive volumes (amounts) of intermediate solutions of cytisine, which are evaporated to dryness before the addition of the respective volumes of the serum pool.

### HPLC Assay

#### Sample preparation

To a 1.5 mL polypropylene tube are applied: 200 µL of serum, 50 µL of working internal standard solution (10 mg/L of sulfanilamide in 0.5 M NaOH), 100 µL of saturated solution of K_2_HPO_4_, and 500 µL of ethyl acetate/acetonitrile mixture (1:1 by vol.); samples are extracted by reciprocating mixing for 20 min, centrifuged at 10 000 g for 5 min, organic supernatant is transferred to a second 1.5 mL polypropylene tube and evaporated to dryness in a vacuum concentration centrifuge for 60 min at 40 °C. Residue is reconstituted with 100 µL of mobile phase, vortex mixed for 15 sec., centrifuged at 10 000 g for 5 min, 80 µL are transferred to 0.25 mL plastic auto-sampler insert, and 50 µL are injected for analysis.

#### Chromatography and quantification

Chromatography is performed using a modular liquid chromatograph consisting of auto-sampler 717 plus, pump 515, column temperature device, photodiode detector 996 and a specialized chromatographic software Empower 32 (Waters Corporation, USA). Separation is achieved on a Hibar RP C18 column (Merck, Purospher STAR, 125 × 4 mm, 5 µm particles), with mobile phase consisting of acetonitrile/0.02 M phosphate buffer, pH 3.0 (2/98 by volume, isocratic flow rate 1.1 mL/min, pressure 1 800 psi), and UV monitoring of the column effluent. Raw data of the chromatograms are collected and processed by Empower software at wavelengths from 220 to 350 nm to assess component peak purities, and quantification is performed at the maximal absorbance of cytisine of 305 nm. All concentrations are calculated in internal standard mode with use of a sevenpoint calibration curve and cytisine/internal standard area ratio as a quantitative measure.

### Method validation strategy

The assay is completely validated using spiked animal serum samples, including selectivity, linearity, intra- and inter-day performance and stability, according to the industrial requirements (Bioanalytical method validation, 2001). Pre-defined acceptance criteria are: 85 to 115% accuracy and precision of the single determinations, no more than two different of six per run should have been out of range. QC-samples are used for calculating accuracy and precision within-run and between runs, as well as recovery of the extraction procedure and stability parameters of the method; 75% or a minimum of 6 standards, when back-calculated should have fallen within ± 15%, except for the lowest limit of quantification (LLOQ), when they should have been within ± 20%; one point with values falling outside these limits could be discarded (except for LLOD and upper limit of quantification, ULOD calibration point), provided it did not change the established model. Acceptance criteria should always be assessed by comparing experimental concentrations against spiked (theoretical) concentrations.

### Experimental animals

Male and female New Zealand rabbits (mean body weight 2.46 ± 0.75 kg) are kept under standard laboratory conditions (20 °C, humidity 60%, cycle – 12 hours light, 12 hours dark), with unrestricted access to granulated standard food and water. The number of animals used for oral and intravenous administration is 8 and 10 rabbits respectively. The trial is performed in compliance with the requirements of European convention for the protection of vertebrate animals used for experimental and other scientific purposes (ETS123, 1991).

### Cytisine administration

#### Intravenous administration (IV)

Cytisine for intravenous administration is dissolved in sterile apyrogenic saline. Intravenous administration is performed in the marginal ear vein of the rabbits at a volume of 0.5 mL/kg b.w. (dose 1 mg/kg b.w.) with sterile disposable syringes and needles.

#### Oral administration (PO)

Cytisine is dissolved in tap water. By means of a plastic catheter the solution is administered directly into the stomach of the animal at a volume of 1 mL/kg b.w. (dose 5 mg/kg b.w.) after an overnight fasting.

All cytisine solutions are prepared ex tempore.

### Blood sampling and processing

Blood samples (volume 1–1.2 mL) are taken from the marginal vein of the other ear at the following intervals – immediately before administration and 0.083, 0.167, 0.333, 0.5, 1, 2, 3, 4 and 6 hours after IV administration and, immediately before administration and 0.5, 1, 1.5, 2, 3, 4, 6 and 8 hours after PO administration respectively. Total blood loss is 10–12 mL per animal. Forty minutes after withdrawal blood is centrifuged for 5 minutes at 10 000 rpm (Eppendorf, MiniPlus centrifuge) to obtain 0.4–0.5 mL serum. Serum is frozen at −20 °C until quantitative analysis of cytisine.

### Calculation of pharmacokinetic parameters

The pharmacokinetic parameters are calculated using TOPFIT (version 2.0) software: AUC_0 − ∞_ (µg*h/L), AUC_0–t_ (µg*h/L), AUMC (µg*h^2^/L), Cl_tot_ (mL/min), C_max_ (µg/L), Cs (µg/L), F (%), MRT_0–t_ (h), MAT (h), t1/2 (h), T_max_ (h), V_d_ (L and L/kg) applying a non-compartmental approach to the experimental cytisine concentrations in blood serum.

Statistical processing of results is performed with the help of MICROSOFT EXCEL 2000 software. Results are expressed as mean values ± SD and compared using Student-Fisher's t-test.

## Results

### HPLC Assay Development

**Figure 1:**
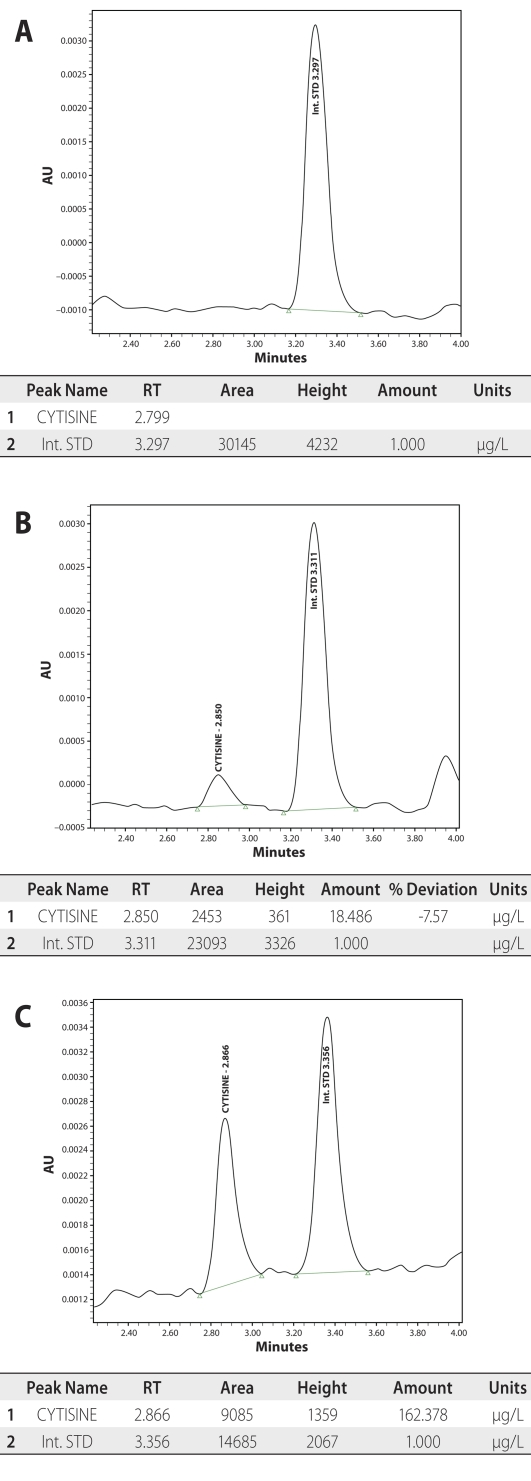
**A**: Zero sample chromatogram. **B:** Chromatogram of low quality control sample. **C:** Chromatogram derived of experimental study animal serum obtained one hour post dose.

Chromatography. Cytisine and internal standard are baseline separated within 4.0 minutes and eluted in the form of symmetric peaks with no interference from biologic matrix. Representative chromatograms are shown in Figure 1.

Selectivity is evaluated by analyzing blank samples (extracted without internal standard), and spiked samples at LLOQ level from five different sources of serum. For each source, the interference is evaluated by comparing the signal in blanks with the signal of the corresponding LLOQ spiked samples. For all blanks, cytisine and internal standard retention windows are free from endogenous interfering peaks. No serum matrix effect is observed under the described extraction procedure.

Accuracy and precision are assessed via the analysis of QC samples at three levels in 6 independently processed batches. Within-run experiments are done together with the experiments for establishment of the LLOQ level for this method. Tables 1 and 2 represent the within-run results, between-run results of the accuracy and precision, as well as the LLOQ results.

**Table 1:** Within-run accuracy and precision for QC samples and LLOD QC sample.

	LLOD = 12.0 µg/L		QC L = 20.0 µg/L		QC M = 1081.0 µg/L		QC H = 2162.16 µg/L	
				
	C, µg/L	d%	C,µg/L	d%	C, µg/L	d%	C, µg/L	d%
**Sample 1**	13.432	11.93	18.622	−6.89	1062.556	−1.71	2222.301	2.78
**Sample 2**	13.468	12.24	19.561	−2.19	1086.629	0.52	2197.365	1.63
**Sample 3**	12.824	6.87	19.420	−2.90	1085.081	0.38	2192.440	1.40
**Sample 4**	11.860	−1.16	20.750	3.75	1044.334	−3.39	2007.323	−7.16
**Sample 5**	11.499	−4.17	20.382	1.91	1083.234	0.21	2358.147	9.06
**Sample 6**	10.916	−9.03	19.123	−4.38	1042.450	−3.57	2145.089	−0.79
**AVERAGE**	12.333		19.643		1067.381		2187.111	
**SD**	1.064		0.973		20.548		113.767	
**CV%**	8.6		4.0		1.9		5.27	

**Table 2:** Between-run accuracy and precision for QC samples.

	QC L = 20.0 µg/L		QC M = 1081.0 µg/L		QC H = 2162.16 µg/L	
			
Date	C,µg/L	d%	C, µg/L	d%	C, µg/L	d%
**06.02.2006**	19.123	−4.38	1042.450	−3.57	2145.089	−0.79
**07.02.2006**	18.486	−7.57	1065.534	−1.43	2144.737	−0.81
**08.02.2006**	20.091	0.45	1083.462	0.23	2196.527	1.59
**09.02.2006**	18.561	−7.20	1017.826	−5.84	2138.821	−1.08
**10.02.2006**	20.819	4.09	1100.598	1.81	2257.346	4.40
**13.02.2006**	19.598	−2.01	989.890	−8.43	2062.041	−4.63
**AVERAGE**	19.446		1049.960		2157.427	
**SD**	0.909		41.537		65.224	
**CV%**	4.7		4.0		3.0	

Extraction recovery is determined by comparing the peak areas from the extracted QC samples with un-extracted standards that represent 100% recovery. The extraction recovery of cytisine is constant for the tested concentration levels (QC low and QC high), averaging 62%.

**Figure 2:**
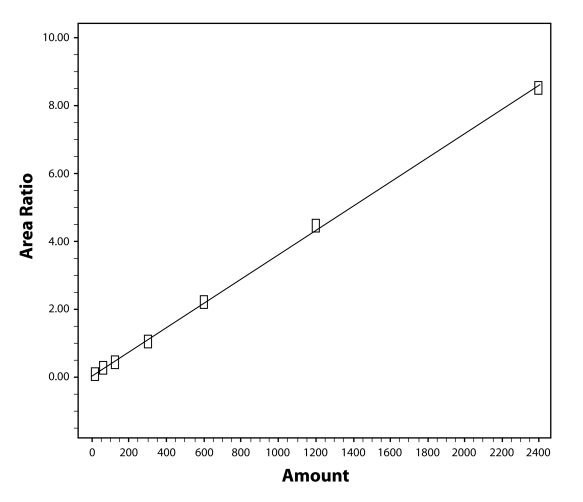
Example calibration curve: plot and calculations obtained according to the method.

The linearity range is set between the LLOQ and the ULOQ representing the lowest and the highest concentration points of the established calibration curves. Calibration is performed with 7 standard points at 12.0 (LLOQ), 60.0, 120.0, 300.0, 600.0, 1 200.0, and 2 400.0 (ULOQ) µg cytisine/L. Calibration curve is generated using the analyte to internal standard peak area ratios by weighted (1/x) least-squares linear regression. The calibration model is selected based on the analysis of the data by linear regression with/without intercepts and weighting factors (1/x, 1/x^2^ and 1/√x). The best fit for the calibration curve is achieved with the linear equation with a 1/x weighting factor. Original results and calibration plot of a calibration curve example are shown in Figure 2.

The lower limit of quantification for cytisine, representing a signal to noise ratio of 10:1 is set at 12.0 µg/L. Results of the experiments validating LLOQ are presented in Table 1. Accuracy and precision are all within the acceptance criteria at this level. The mean response for cytisine peak at the assay LLOQ is about 10-fold grater than the mean response for the five blanks at the peak retention time. The limit of detection (LOD) for this method is around 4.0 µg/L of cytisine in extracted/injected samples with mean response that is over 3-fold greater than the mean response for the five blank samples at the peak retention time.

Stability studies. Freeze and thaw stability is determined after three freeze and thaw cycles each lasting 24 h. Quality control samples are extracted and analyzed after the third cycle (two levels in duplicate), and results are compared to the theoretical (spiked) values. Short-term stability is validated for 6 h and post-preparative stability is validated for 24 h, both at room temperature. Long term stability is evaluated by the analysis of quality control samples on three different occasions within 95 days. Stock solution stability is evaluated by direct analysis of the working standard in mobile phase on three different occasions in the respective three months time frame. Results of the stability experiments are always well within ± 15% of deviation, and thus it is proven that no degradation of cytisine occurred under the conditions described.

Analysis of study samples started after the time of last blood sampling point of the experimental animals and is performed in several consecutive runs, each encompassing its own calibration curve and set of QC samples. After completion of each chromatographic run chromatograms are inspected for correct integration without knowing the respective concentration. Concentrations of unknown study samples are only calculated if results of CC and QC samples for each run met the pre-defined acceptance criteria.

### Pharmacokinetics

**Figure 3:**
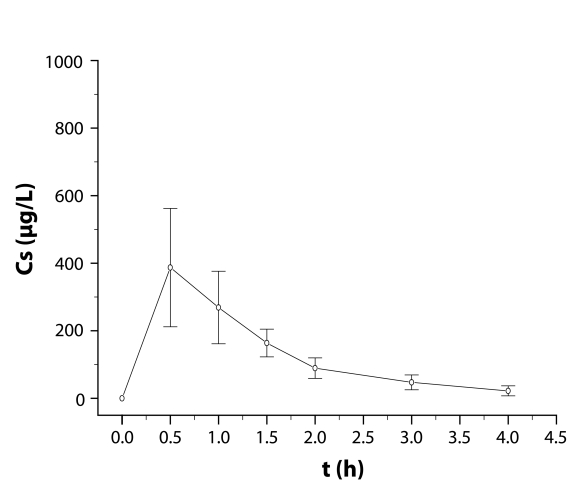
Cytisine serum concentrations (Cs in µg/L) after oral administration in rabbits (dose 5 mg/kg b.w.) (mean values ± SD, n = 8).

**Figure 4:**
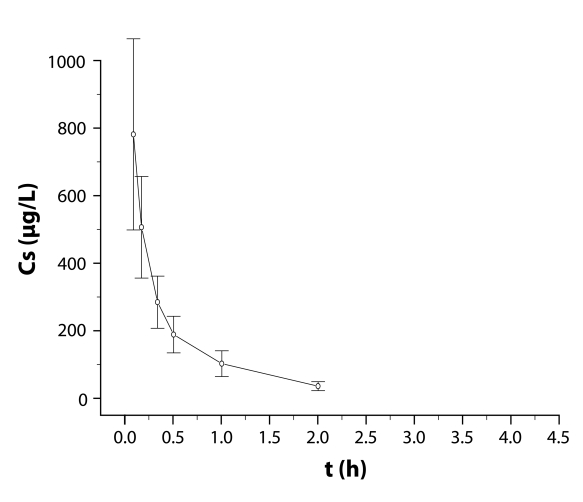
Cytisine serum concentrations (Cs in µg/L) after intravenous administration in rabbits (dose 1 mg/kg b.w.) (mean values ± SD, n = 10).

Serum concentrations determined in the course of the trial are given in Tables 3 and 4 and Figures 3 and 4 for oral and intravenous administration of cytisine respectively.

**Table 3:** Serum concentration (mg/L) of cytisine after oral administration in rabbits (dose 5 mg/kg b.w.), (n = 8).

t (h)	0	0.5	1.0	1.5	2.0	3.0	4.0	6.0	8.0
**Rabbit A**	0	592.77	375.26	180.14	109.96	85.43	28.90	BDM	BDM
**Rabbit B**	0	146.38	100.86	131.51	59.494	23.39	87.97	BDM	BDM
**Rabbit C**	0	411.51	315.84	137.51	64.702	37.08	14.35	BDM	BDM
**Rabbit D**	0	563.66	425.13	229.36	152.08	73.06	52.46	BDM	BDM
**Rabbit E**	0	245.63	259.12	153.21	75.05	39.98	14.32	BDM	BDM
**Rabbit F**	0	409.80	277.28	152.33	84.25	45.75	15.53	BDM	BDM
**Rabbit G**	0	538.87	246.48	214.87	98.01	24.87	15.70	BDM	BDM
**Rabbit H**	0	188.95	151.94	113.05	72.83	49.40	14.98	BDM	BDM

**MEAN**	0	387.196	268.989	163.997	89.547	47.371	30.526	–	–
**SD**		175.191	107.243	40.929	30.323	21.900	26.712	–	–

**Table 4:** Serum concentration (mg/L) of cytisine after intravenous administration in rabbits (dose 1 mg/kg b.w.), (n = 10).

t (h)	0.0833	0.167	0.333	0.5	1.0	2.0	3.0	4.0	6.0
**Rabbit I**	1063.47	754.36	386.45	239.33	123.45	26.38	43.38	BDM	BDM
**Rabbit J**	790.03	491.72	274.95	197.02	148.09	31.89	BDM	BDM	BDM
**Rabbit K**	680.34	570.97	301.94	229.01	125.02	24.53	BDM	BDM	BDM
**Rabbit L**	810.53	465.82	286.82	141.85	84.09	BDM	BDM	BDM	BDM
**Rabbit M**	1282.30	526.09	345.66	222.36	127.14	30.14	29.65	BDM	BDM
**Rabbit N**	504.15	232.30	136.37	99.54	39.29	BDM	BDM	BDM	BDM
**Rabbit O**	417.78	303.56	169.64	108.42	44.74	22.28	BDM	BDM	BDM
**Rabbit P**	BDM	629.29	336.45	196.15	140.32	22.72	BDM	BDM	BDM
**Rabbit Q**	BDM	563.96	304.67	257.25	115.25	58.99	20.11	BDM	BDM
**Rabbit R**	695.11	525.41	310.64	200.00	91.43	46.02	BDM	BDM	BDM

**MEAN**	780.465	506.348	285.358	189.093	103.883	37.503	31.048	–	–
**SD**	282.367	150.168	77.064	54.631	38.006	13.041	11.699	–	–

The pharmacokinetic behaviour of cytisine after oral and intravenous administration is evaluated. The values of the following pharmacokinetic parameters are calculated: T_max_, C_max_, AUC_0–t_, AUC_0 − ∞_, AUC_t*−*∞_, F, t1/2, Vd, Cl_*tot*_, AUMC_0*–t*_ and MRT_*0–t*_. (The values are given as means ± standard deviation).

**Table 5:** Pharmacokinetic parameters of cytisine after oral (dose 5 mg/kg b.w.) and intravenous administration (dose 1 mg/kg b.w.). Mean values (SD). Relative bioavailability F (%) after oral administration is calculated by the equation: F = 100*AUC_po_/5*AUC_iv_

Parameter	Oral administration Mean values (SD)	Intravenous administration Mean values (SD)
**T_max_ (h)**	0.59 (0.19)	–
**C_max_ (µg/L)**	388.89 (173.69)	–
AUC_0–t_(µg*h/L)	542.80 (207.78)	319.68 (105.39)
AUC_0−∞_ (µg*h/L)	563.92 (211.15)	350.48 (111.99)
AUMC_0–t_ (µg*h^2^/L)	707.96 (338.89)	189.20 (95.48)
**MRT_0–t_ (h)**	1.30 (0.15)	0.56 (0.15)
**t_1/2_ (h)**	0.86 (0.17)	0.61 (0.19)
**V_d_ (L)**	13.06 (7.07)	2.55 (0.89)
**V_d_ (L/kg)**	6.21 (2.92)	1.02 (0.47)
**Cl_tot_ (mL/min)**	167.2 (64.68)	42.99 (23.60)
**F_0−∞_ (%)**	32.18	–
**F_0−t_ (%)**	33.96	–

## Discussion

Cytisine is among the oldest means to treat tobacco dependence. After more than 40 years on the market, cytisine still is of considerable interest mainly because of its high efficacy and safety and low cost. Selective α_4_β_2_-nicotinic acetylcholine receptor ligands are important in the search for medicines for the treatment of tobacco dependence. Cytisine is an agonist of α_4_β_2_-nicotinic receptors with a high affinity for receptors (Ki = 0.45 nM) (Gonzales *et al*., 2006, Jorenby *et al*., 2006, Tonstad *et al*., 2006). There are data in the literature (Coe *et al*., 2005, Imming *et al*., 2001) suggesting that cytisine affinity for these receptors is 7 times higher than that of nicotine.

Unfortunately cytisine pharmacokinetics in laboratory animals are scarce and disposition in man is practically not studied at all. This is the reason why the study of cytisine kinetics in animals is relevant.

The developed HPLC analytical method for the assay of cytisine in blood serum is highly selective and sensitive. Linearity of the method is in the range 12–2 400 µg/L; accuracy and precision are both within ± 10%, and the limit of detection is 4 µg/L. It proves suitable for the purpose of the study of cytisine pharmacokinetic behaviour.

The ratio AUC_0–t_/ is less than 5% (4.46 ± 2.28) for the oral administration and less than 10% (9.24 ± 4.35) for the intravenous administration. That proves that the time interval for measuring serum levels are properly chosen. Maximum serum concentrations after oral administration are observed within 35 minutes on the average, which suggests a rapid absorption of cytisine in gastrointestinal tract. C_max_ is 388.9 ± 173.69 (µg/L). The rate of elimination of cytisine is relatively fast – the t_1/2_ is 51 min for the oral and 36 min for the intravenous administration. Sariev *et al*., (1999) also found a half life of less than an hour (57 min) in the same animal model. The total body clearance found in our animal model (42.99 ± 23.6 mL/min) after intravenous application is practically the same as the value found by the same authors – 40 mL/min.

The ratio F between AUC after oral and AUC intravenous administration (corrected for the dose) is 32.18% suggesting an incomplete absorption or first pass metabolism. The knowledge of cytisine kinetics would prove useful for the development of new pharmaceutical formulations with modified release which could improve user's compliance.
